# Associated screening for HCV and SARS‐Cov2 infection in an urban area of Southern Italy: A cohort study

**DOI:** 10.1111/jvh.13623

**Published:** 2021-10-06

**Authors:** Carmine Coppola, Mario Masarone, Marco Bartoli, Laura Staiano, Roberta Coppola, Pietro Torre, Massimiliano Conforti, Daniela Amoruso, Ivan Gardini, Marcello Persico

**Affiliations:** ^1^ Department of Internal Medicine ‐ Unit of Hepatology and Interventional Ultrasonography OORR Area Stabiese Plesso Nuovo Gragnano Gragnano Italy; ^2^ Internal Medicine and Hepatology Division Department of Medicine and Surgery Scuola Medica Salernitana” University of Salerno Baronissi Italy; ^3^ EpaC Onlus Italian Liver Patient Association Turin Italy

**Keywords:** case‐finding, epidemiology, HCV infection, SARS‐CoV2 pandemic

## 1 SHORT COMMUNICATION

In Italy, since the first months of 2020, the COVID‐19 pandemic has led the government to set a series of strict rules limiting all the non‐essential clinical activities.^1^ These measures reduced the resources for managing potentially curable diseases such as HCV infection. As a matter of fact, this negative impact has been demonstrated by a recent survey carried out by the Italian Association for the Study of the Liver (AISF) where a nearly total interruption of HCV therapies during the first lockdown phase was reported.^2^ In addition to that, the principal barriers to obtaining HCV elimination are related to the actual ability to reach all infected patients rather than to the effectiveness of the therapy, as it has been reported that only 20% of the HCV‐infected subjects have been already diagnosed worldwide.^3^ In Italy, the latest available data estimate that there is 20% of undiagnosed subjects, as well as that 50% of diagnosed patients, are still awaiting a cure.^4^ Moreover, updated data on the real prevalence in the Italian general population are scarce and incomplete.^4^ Finally, many speculations have been made about the effective number of SARS‐CoV2 asymptomatic carriers and their importance on its spreading across the globe.^5^ However, there is limited evidence on this burden, also because universal screening for SARS‐CoV2 has not been initiated yet. Here we report the results of an associated screening program for SARS‐CoV2 and HCV infection on the whole available population of a small town, to be used as a representative cohort of a Southern Italy population, aimed at finding out the real prevalence of both diseases.

## 2 PATIENTS AND METHODS

A prospective observational cohort study was set up with the aim of testing for both SARS‐CoV2 and anti‐HCV antibodies using rapid blood tests in all the available populations of Casola Di Napoli, a small town in the southern province of Naples. This town has been specifically chosen because it is in the Campania region, previously reported as one of the areas with the highest prevalence of HCV, but also with the higher treated and untreated patients’ ratio in Italy. From a total of 3,845 inhabitants, 3,556 were considered eligible, after excluding 225 subjects of age <6 years and 64 who were resident abroad at the time. Of them, 2,740 (77.05%) participated voluntarily after signing informed consent. The other 816 subjects (22.95%) did not respond to the repeated public appeals made through advertising on the local media and social networks, by information posters in commercial activities, meeting places, at GPs and pharmacies by the municipal administration throughout the screening period. No one refused to give informed consent. The study was approved by our local Ethics Committee (Comitato Etico Campania Sud). Each enrolled subject underwent a rapid blood‐spot test for anti‐HCV with an Advanced Quality™ Rapid Anti‐HCV Test (InTec Products, Inc.), and for IgM and IgG anti‐SARS‐CoV2 (SARS‐CoV‐2 IgM/IgG combined antibody rapid test kit, VivaDiag, VivaChek Biotech).^6^ All patients who tested positive to SARS‐CoV2 antibodies underwent a nasopharyngeal swab to perform an Allplex 2019‐nCoV assay (Seegene Inc.) used on a Nimbus IVD and Cfx‐96™ Real‐time PCR automatic extractor(Seegene Inc.).^7^ All anti‐HCV‐positive patients underwent a standard serum sampling for AST and ALT, anti‐HCV and HCV RNA assaying by PCR (Cobas TaqMan v2.0, CAP/CTM HCV v2, Roche Molecular Systems, Pleasanton, CA). Moreover, a brief questionnaire was given to all participants, asking if they were aware of an HCV infection and, if so, if they ever underwent any antiviral therapy and what was the known outcome.

## 3 RESULTS

The screening was carried out from June 25 to July 12, 2020. A total of 39 patients (1.4% of the total) were positive for either SARS‐CoV2 IgM or IgG. None of these patients were positive for SARS‐CoV2‐RNA nor anyone reported symptoms suggesting an actual/previous COVID‐19 disease. Fifty‐four patients (1.97% of the total) were positive for anti‐HCV by a rapid blood test. These patients underwent a confirmatory serum anti‐HCV test with a commercial ELISA assay. Thirteen patients (13/54; 24.07%) were negative in the confirmatory test, therefore false‐positive with the rapid blood‐test (Table 1). In only one case a combined positivity for SARS‐CoV‐2 and HCV was found. The only significant difference between anti‐HCV‐positive and anti‐HCV‐negative subjects was an older age in HCV‐positive. Of the 41 patients confirmed anti‐HCV‐positive, 36 (87.8%) reported awareness of their status and, in 86.1% (*n* = 31), reported successful antiviral treatment in the past. Of the remaining four patients aware of their positivity, two were HCV RNA‐positive (one never undergone an antiviral treatment), whereas the other two were spontaneously negative. The remaining five anti‐HCV‐positive patients were unaware of their positivity. Of them, two patients were HCV RNA‐negative and three were positive (Table 1). Antiviral treatment received was with IFN containing regimens (45.1%) or IFN‐Free DAAs (54.8%). Only one patient was unsuccessfully treated with standard Interferon in the past, and still HCV RNA positive to date, therefore with an overall Sustained Virological Response of 96.87%. The anti‐HCV positivity was only detected in patients 41 years or older. Of notice, the most conspicuous number of anti‐HCV‐positive patients was in the 61–80 years age class with a total of 25/41 (60.98%). Also, the overall prevalence was higher in older age groups in respect to the others, with 32 patients over a total of 904 aged 61–80 (5.3%) and 4/69 (5.8%) aged >81 years resulting positive with the rapid HCV test (Table 1). Therefore, from the data obtained by the present screening, the seroprevalence of anti‐SARS‐CoV2 and anti‐HCV was 1.4% and 1.49%, respectively. No active infections of SARS‐CoV2 were detected, whereas five patients (0.18%) had an active HCV infection.

**TABLE 1:** Overall characteristics of study population

Variable	Overall (% on overall)	quick HCV‐Ab negative (%)	quick HCV‐Ab positive (%)	*p*
*N* (%)	2738 (100%)	2684 (98.03%)	54 (1.97%)	
Age mean (SD)	45.57 (19.45)	45.19 (19.34)	64.46 (14.73)	<0.0001
Sex % (M/F)	45.5/54.5	45.8/54.2	33.3/66.7	0.074
Not Italian (%)	0.6% (n16)	0.59%	0	0.56
HCV rapid test positive	54 (1.97%)	‐	54	‐
HCVAb confirmation positive	41 (1.49%)	‐	41	‐
HCV Already Known	36/41(87.8%)	‐	36/54 (66.67%)	
HCVRNA positive	5 (0.18%)	‐	5/54 (9.26%)	‐
HCVRNA positivity not already known	3 (0.11%)	‐	3/54 (5.55%)	
SARS‐COV2 Ab positive	39 (1.4%)	38 (1.41%)	1/54 (1.9%)	0.54
IgM+IgG	17 (0.6%)	16 (0.59%)	1/54 (1.9%)	0.75
IgM	20 (0.7%)	20 (0.74%)	0	0.53
IgG	2 (0.1%)	2 (0.07%)	0	0.17
SARS‐Cov2 NS swabs (PCR)	0	0	0	‐
Age classes	Overall population	SARS‐Cov2 IgM/IgG rapid blood test positive	HCV‐Ab quick blood test positive	
<20 years	348 (12.7%)	0 (0%)	1 (0.3%)	
21–40 years	721 (26.3%)	2 (0.3%)	2 (0.3%)	
41–60 years	996 (36.4%)	14 (1.4%)	14 (1.4%)	
61–80 years	604 (22.1%)	20 (3.3%)	33 (5.5%)	
>81 years	69 (2.5%)	3 (1.4%)	4 (5.8%)	

Note

No statistically significant differences, other than older age for HCV positive, were found between HCV‐Ab positive and negative subjects at the blood quick test.

Abbreviations: Ab, antibodies; NS, nasopharyngeal

## 4 DISCUSSION

Even though the SARS‐CoV2 pandemic has profoundly influenced the diagnosis and the cure of HCV infection, against all odds, recent estimates tell us that Italy could still be on track to reach HCV elimination by 2030.^8^ However, the major obstacles to obtain this goal are the uncertainty on the data of HCV prevalence, the unawareness of infection by a percentage of subjects and the lack of linkage‐to‐care for those aware. On the other hand, little is known about the burden of asymptomatic infections by SARS‐CoV2 in Italy. Our data suggest that anti‐HCV prevalence is 1.49% and, in line with the previsions of previous reports, in decline in respect to older epidemiological data. Moreover, the age distribution of anti‐HCV‐positive subjects further confirms that HCV infection in the general population is an almost exclusive prerogative of older subjects. Optimistically, only 0.18% of the study population had an active HCV infection and, of these, three patients were unaware of their infection. This good finding was partially due to the fact that the majority (88.6%) of patients who were aware of their infection had already been treated in the past. More than a half of them (53%) has been subjected to antiviral treatment in recent times, also thanks to the efforts of the nearby Gragnano Hospital Hepatology team, which treated more than 2,600 patients with DAA. The other likely cause of such a low prevalence is probably to be found in the characteristics of the study cohort, which is a close community of residential workers, without any significant exchange and/or drug abuse problem in the small town. In this way, the high‐risk/high‐prevalence populations are very likely little represented in this cohort. Nevertheless, it must be pointed out that those five patients with active infection represent the 12.2% of anti‐HCV‐positive subjects, and the three patients unaware of their active infection were the 60% of the total active cases, highlighting that the efforts of case‐findings cannot be reduced. Moreover, during our screening, we found 13 anti‐HCV false‐positives, a very high percentage from 54 subjects (24.07%). The most plausible explanation could be that the health workers performing the screening were instructed to “consider as positive” also the tests that could have been labelled as “uncertain.” However, it must be noted that a higher rate of false‐positive should very likely not impact the sensitivity of the test, which is reported to be as high as 99.0% (CI:96.5%–99.7%), the most important parameter to pay attention to for screening purposes. Finally, the SARS‐CoV2 screening revealed that in a lower incidence period for Covid‐19 in Italy, only 39/2738 subjects(1.4%) showed a previous, asymptomatic contact with SARS‐CoV2, no one being actively infected at the moment of the screening, neither reporting a symptomatic infection in the past. This low prevalence can be explained also by the fact that Southern Italy was, *de facto*, partially left untouched by the first wave of the epidemic. In conclusion, our study shows that during a low incidence period, previous asymptomatic contact with SARS‐CoV2 was 1.4%. HCV infection was present in 1.49% of the subjects tested, of whom 12.2% had an active infection which in 60% of cases was not known. This study shows that HCV infection is reducing in prevalence, especially in regards to active infection, thanks to the antiviral therapy programs and that, in the general population, it is restricted to the older subjects. Finally, it demonstrates that also during the pandemic there may be the opportunity to promote prevention, disseminate information, and perform screening activities for HCV infection, *n* an attempt to reach the achievable goal of HCV elimination by 2030.

## CONFLICT OF INTEREST

The authors declare no conflict of interest for the present work.

## Data Availability

Study data will be given upon reasonable request made to the corresponding author (Prof. Marcello Persico: mpersico@unisa.it).

## References

[1] Organization WH . WHO Timeline ‐ COVID‐19. 2020; https://www.who.int/news‐room/detail/27‐04‐2020‐who‐timeline‐‐‐covid‐19. Accessed June 13, 2020.

[2] Aghemo A , Masarone M , Montagnese S , et al. Assessing the impact of COVID‐19 on the management of patients with liver diseases: a national survey by the Italian association for the study of the Liver. Dig Liver Dis. 2020;52(9):937‐941. 32703730 10.1016/j.dld.2020.07.008 PMC7351426

[3] WHO . Global hepatitis report, 2017. [Pdf]. 2017; https://apps.who.int/iris/bitstream/handle/10665/255016/9789241565455‐eng.pdf. Accessed December, 31, 2020.

[4] Gardini I , Bartoli M , Conforti M , Mennini FS , Marcellusi A . Estimation of the number of HCV‐positive patients in Italy. PLoS One. 2019;14(10):e0223668. 31671120 10.1371/journal.pone.0223668 PMC6822946

[5] Gao Z , Xu Y , Sun C , et al. A systematic review of asymptomatic infections with COVID‐19. J Microbiol Immunol Infect. 2020:54(1):12‐16. 32425996 10.1016/j.jmii.2020.05.001 PMC7227597

[6] Paradiso AV , De Summa S , Loconsole D , et al. Rapid serological assays and SARS‐CoV‐2 real‐time polymerase chain reaction assays for the detection of SARS‐CoV‐2: comparative study. J Med Internet Res. 2020;22(10):e19152. 33031048 10.2196/19152 PMC7641647

[7] Farfour E , Lesprit P , Visseaux B , et al. The Allplex 2019‐nCoV (Seegene) assay: which performances are for SARS‐CoV‐2 infection diagnosis? Eur J Clin Microbiol Infect Dis. 2020;39(10):1997–2000. 32462501 10.1007/s10096-020-03930-8 PMC8824685

[8] Razavi H , Sanchez Gonzalez Y , Yuen C , Cornberg M . Global timing of hepatitis C virus elimination in high‐income countries. Liver Int. 2020;40(3):522‐529. 31815353 10.1111/liv.14324

